# YTHDF1/RNF7/p27 axis promotes prostate cancer progression

**DOI:** 10.1038/s41419-025-07648-3

**Published:** 2025-04-18

**Authors:** Yulin Shi, Baiyang Liu, Yong Zhang, Sen Zhao, Li Zuo, Jun Pu, Haoqing Zhai, Dengcai Mu, Jia Du, Yan Cheng, Cuiping Yang, Yongbin Chen

**Affiliations:** 1 The First Affiliated Hospital of Zhengzhou University, Zhengzhou, China; 2 State Key Laboratory of Genetic Evolution & Animal Models, Kunming Institute of Zoology, Chinese Academy of Sciences, Kunming, Yunnan China; 3 Department of Neurosurgery, The Second Affiliated Hospital of Kunming Medical University, Kunming, Yunnan China; 4 Department of Pathology, Cancer Hospital of China Medical University, Shenyang, Liaoning China; 5 The Affiliated Changzhou Second People’s Hospital of Nanjing Medical University, Changzhou Second People’s Hospital, Changzhou Medical Center, Nanjing Medical University, Nanjing, China; 6 The International Peace Maternity and Child Health Hospital, School of Medicine, Shanghai Jiao Tong University, Shanghai, China; 7 Shanghai Key Laboratory of Embryo Original Diseases, Shanghai, China

**Keywords:** Tumour biomarkers, Oncogenesis

## Abstract

Prostate cancer (PCa) is a prevalent malignant tumor of the urinary system and remains the most common cancer among males. In this study, we showed that YTHDF1, one of the reader proteins involved in the N6-methyladenosine (m6A) modification signaling pathway, is highly expressed in PCa cancerous tissues and cells, which correlates with poor clinical outcomes. Our study revealed that YTHDF1 knockdown inhibits tumor cell proliferation, migration, and xenograft tumor formation by decreasing p27 protein stability through proteasome degradation signaling. Consistently, YTHDF1 depletion markedly reduced the clonogenic growth of *Pten* or/and *TP53*-deficient organoids. Candidate p27-targeting E3 ubiquitin ligases screening identified RNF7 as the direct downstream target for YTHDF1 in an m6A-dependent manner. The subsequent high translation of RNF7 results in the efficient degradation of the cell cycle inhibitor p27 and malignant tumor cell growth. In addition, we provided evidence showing that YTHDF1 or RNF7 depletion sensitizes tumor cells to chemotherapy drug cisplatin by increasing cellular apoptosis. Our findings revealed that the neddylation inhibitor MLN4924 effectively inhibited prostate cancer progression in vitro and in vivo. Our study highlights the YTHDF1/RNF7/p27 axis as a crucial component in PCa, suggesting its potential as a novel therapeutic target.

## Introduction

Prostate cancer (PCa) is a prevalent malignant tumor in the urinary system, and it remains the most common male tumor, according to the latest cancer statistics in the United States [1]. Prostate cancer alone accounts for one-fifth of newly diagnosed cases, and the death rate of prostate cancer also ranks second among male tumors [1]. Older age, genetic mutations, diet, and inflammation are significant factors in the etiology of PCa [2]. Previous reports have shown that PCa is rare in eastern countries due to the diet habit dominated by plants [2]. However, based on the statistical data of cancer in China from 2000 to 2011, with the improvement of living standards in our country, the aggravation of environmental pollution, and the gradual westernization of dietary structure, the incidence and mortality of prostate cancer have also increased significantly [3].

Prostate cancer diagnosis involves prostate biopsy, supported by prostate-specific antigen (PSA) testing, magnetic resonance imaging (MRI), digital rectal examination, and/or health screening. However, due to the subtlety of early symptoms in prostate cancer, the disease is frequently not detected until it has progressed to the middle or late stages, when symptoms become more apparent. Therefore, early screening for prostate cancer holds significant importance [4–7]. Currently, the clinical approaches for the treatment of prostate cancer primarily encompass surgical intervention, radiotherapy, chemotherapy, and endocrine therapy, among others [8]. The standard primary therapy for metastatic PCa is androgen deprivation therapy (ADT) by surgical or medical castration [2]. Despite the above available treatment options, the therapeutic effects are still greatly constrained, drug- and radio-resistance will almost inevitably occur, which eventually leads to further deterioration and metastasis of tumors. The discovery of novel cancer biomarkers holds potential for the targeted treatment of prostate cancer (PCa). A growing body of evidence highlights the genetic contributions to prostate cancer, including identifying common genes that function as biomarkers for this disease, including BRCA genes, RNase L (HPC1, lq22), HOX genes, and the ATM gene [8]. Mutations in androgen receptors have been associated with the emergence of androgen resistance receptor-targeted therapies [9]. Malfunctions in the signaling pathways, including the MAPK/ERK pathway, AR (androgen receptor) related pathways, and the contribution of Akt/PI3K pathways to ADT resistance have been established[10]. Biomarkers are anticipated to delineate precise treatment specifications for cancer. However, existing clinical therapies tend to benefit only a limited subset of patients and are often associated with numerous side effects that significantly diminish the quality of life for the majority of patients. Consequently, there remains an urgent necessity to screen novel molecules that influence the onset and progression of prostate cancer, as well as to elucidate their functions and underlying mechanisms, to develop effective treatments for prostate cancer in the future.

Recently, our research group has developed innovative strategies for identifying novel biomarkers associated with mammalian hypoxia adaptation and hypoxic solid tumors [11] and a normalization-free methodology exploring pan-cancer biomarkers [12]. Our study identified YTHDF1, a reader protein in the N6-methyladenosine (m6A) modification pathway, as a crucial regulator in non-small cell lung cancer (NSCLC) [11]. m6A modification influences RNA post-transcriptional processes, including splicing, stability, and protein translation, thereby regulating crucial biological activities such as hematopoietic stem cell differentiation, spermatogenesis, and central nervous system development. It also plays a role in the progression of various human diseases, including cancers, diabetes, and infectious diseases [13, 14]. The key factors involved in RNA-m6A modification are divided into three categories: m6A methyltransferase (Writer), including METTL3, METTL14, WTAP, RBM15/15B, KIAA1429, etc.[15–19].; m6A demethylase (Eraser), including ALKB dioxygenase family members FTO and ALKBH5 [20]; m6A binding protein (Reader), including YTH domain family protein members YTHDF1, YTHDF2, YTHDF3, YTHDC2, YTHDC1 family proteins, IGF2BP family proteins and HNRNP, etc. Recent research has demonstrated a significant association between m6A modification and various aspects of tumor biology, including proliferation, differentiation, invasion, and metastasis [14]. Our findings suggest that YTHDF1 does not enhance prostate cancer progression through the m6A-modified *KEAP1* and Nrf2 axis, as observed in non-small cell lung cancer, highlighting its context-dependent function across different cancer types. This study revealed that YTHDF1 overexpressed in prostate cancer cells and tissues. Moreover, YTHDF1 depletion decreased cell proliferation, migration, and xenograft tumor formation capacity. To investigate the potential therapeutic implications of YTHDF1, we subsequently aimed to examine its functional role and elucidate the underlying mechanisms through which YTHDF1 facilitates the progression of prostate cancer.

## Materials and methods

### Cell culture

HEK293T cells, obtained from the American Type Culture Collection (ATCC), were cultured in Dulbecco’s Modified Eagle’s Medium. Similarly, PC-3, DU145, LNCap, and 22RV-1 cell lines, sourced from ATCC, were cultured in RPMI 1640 medium (Gibco). Both media were enriched with 10% fetal bovine serum (Gibco) and 1% penicillin-streptomycin solution. RWPE-1 cells were maintained in Gibco’s Keratinocyte Serum-Free Medium (K-SFM). Cell culture plates were procured from NEST. All cell lines were cultured consistently in a humidified 37°C environment using a Thermo Scientific CO_2_ incubator.

### Constructs, transfection, and lenti-viral infection

Independent short hairpin RNAs (shRNAs) targeting YTHDF1 were engineered utilizing the pLKO.1 vector and all the constructs were sequence verified. The coding sequences of human YTHDF1 were inserted into the pCDH-CMV-MCS-EF1-copGFP-T2A-Puro vector through standard molecular cloning techniques to produce Flag-tagged fusion proteins. Lentiviruses were subsequently generated following the manufacturer’s protocol. Additionally, we constructed an overexpression plasmid targeting RNF7 using the pBabe-Flag-puro vector, followed by sequencing verification of the plasmid. Specifically, the coding sequence of RNF7 was inserted into the pBabe-Flag-puro vector to produce a fusion protein with a Flag-tag. The pBabe-RNF7-Flag-puro and pCL-Eco plasmids were co-transfected into HEK-293T cells, and the retroviral particles were generated according to the manufacturer’s protocol. Cells were infected with the viral supernatants that contained 4 µg/ml polybrene, in two separate instances, each lasting 48 and 72 h, respectively.

### Cell growth

Cells were maintained in a serum-containing medium for 24 h. Twenty minutes before fixation, the cells were exposed to 10 µM BrdU. They were then fixed with 4% paraformaldehyde (PFA) for 20 min. Cells were stained with a primary antibody targeting BrdU (Abcam, Catalog # ab6326, 1:1000 dilution), followed by a secondary antibody. Nuclear staining was performed using DAPI. Imaging and quantification were conducted on a Nikon Ti fluorescence microscope, with five randomly selected fields per sample being analyzed.

### RIP assay

Cells were lysed for RNA immunoprecipitation (RIP) assays to assess YTHDF1 binding capacity. The RIP assays were performed using the Magna RIP™ RNA-Binding Protein Immunoprecipitation Kit from Sigma-Aldrich (St. Louis, MO, USA). The antibodies employed in the RIP assay included YTHDF1 (Cell Signaling Technology, catalog number 77422 s, dilution 1:20) and IgG (Sigma-Aldrich).

### Polysome profiling

Following the established protocol, 100 μg/ml cycloheximide was introduced into the cell culture medium for 5 min before cell collection. Cells were collected, washed, and lysed with a buffer containing 5 mM Tris-HCl (pH 7.5), 2.5 mM MgCl2, 1.5 mM KCl, 100 μg/ml cycloheximide, 2 mM DTT, 0.5% Triton X-100, 0.5% Sodium Deoxycholate, 200 U/ml RNase inhibitor, and a 1x EDTA-free protease inhibitor cocktail. The resulting cell supernatants were fractionated into 42 fractions, each with a volume of 1 ml, and subsequently analyzed for OD_260_ using a NanoDrop spectrophotometer (Thermo Fisher Scientific). Each fraction sample underwent qRT-PCR to detect the relative mRNA expression levels of p27.

### Immunoprecipitation, immunoblot, and qRT-PCR assays

Cells designated for the immunoprecipitation assay were collected and lysed using RIPA buffer (Beyotime) with added protease inhibitors (Roche). The lysates underwent a 30 min incubation on ice, followed by clarification by centrifugation at 21,500 rpm and 4 °C for 30 min. The protein lysate underwent overnight immunoprecipitation at 4 °C using the primary antibody. The immunoprecipitates were thoroughly washed with ice-cold IP buffer, denatured by boiling in SDS loading buffer, and analyzed via immunoblotting. Total RNA was extracted from the specified cells using RNAiso Plus (Takara) for the qRT-PCR assay. The RNA was then reverse-transcribed into complementary DNA (cDNA) using the PrimeScript RT Reagent Kit (VAZYME). The resulting cDNA was then analyzed via RT-qPCR employing the FastStart Universal SYBR Green Master Mix (VAZYME). All reactions were conducted in triplicate using the Applied Biosystems 7500 instrument. Expression levels were normalized to β-actin. The information on the antibodies, primers, and oligos employed in this study is provided in Tables S1 and S2.

### Xenograft tumor model

Six-week-old male nude mice were randomly divided into experimental groups and subcutaneously injected with either two independent YTHDF1 knockdown cell lines or scramble control shRNA cells, each at a concentration of 1 × 10^6 cells per mouse. The mice were observed daily, with xenograft tumor weights and volumes measured every two days using a sliding caliper. Tumor volumes were determined using the formula (Length × Width²) / 2. After the experiment, all mice were euthanized, and their tumors were surgically removed and weighed. In the xenograft tumor cisplatin sensitivity assay, mice with tumors reaching 50 mm³ received intraperitoneal cisplatin (DDP) injections at 7 mg/kg every 6 days. After four weeks, all mice were euthanized, and their tumors were excised and weighed for analysis.

### Immunohistochemical staining (IHC)

The indicated tumors were excised and subjected to overnight fixation in 4% paraformaldehyde. Subsequently, the tumor tissues were embedded in paraffin and sectioned serially. In summary, the paraffin-embedded sections were subjected to a deparaffinization process utilizing xylene, followed by rehydration through a graded ethanol series, and antigen retrieval was performed. Treatment with hydrogen peroxide (H_2_O_2_) was immediately followed by blocking with 10% normal goat serum. The slide was incubated with the specified primary antibody at 4 °C overnight. After incubation, the slides underwent three washes with PBST. Subsequently, the sections were treated with an HRP-conjugated secondary antibody (DAKO, Denmark) for 40 min. Diaminobenzidine was employed as a chromogen, followed by counterstaining with hematoxylin. The slides were then dehydrated, cleared using xylene, and mounted for analysis.

### Bioinformatics assay and statistical analyses

All datasets utilized in this study are publicly accessible. The mRNA expression data were acquired from the TCGA and GTEx datasets via their official websites. Subsequent analyses were performed using the R programming language and the TNMplot platform. Survival analyses were performed using the GEPIA platform and R, while the Student’s t-test evaluated statistical significance between the two experimental groups. A one-way ANOVA was conducted to compare multiple groups. Statistical significance was indicated by *P*-values: < 0.05 (*), < 0.01 (**), and < 0.001 (***).

## Result

### YTHDF1 is highly expressed in prostate cancer cells

**Fig. 1:**
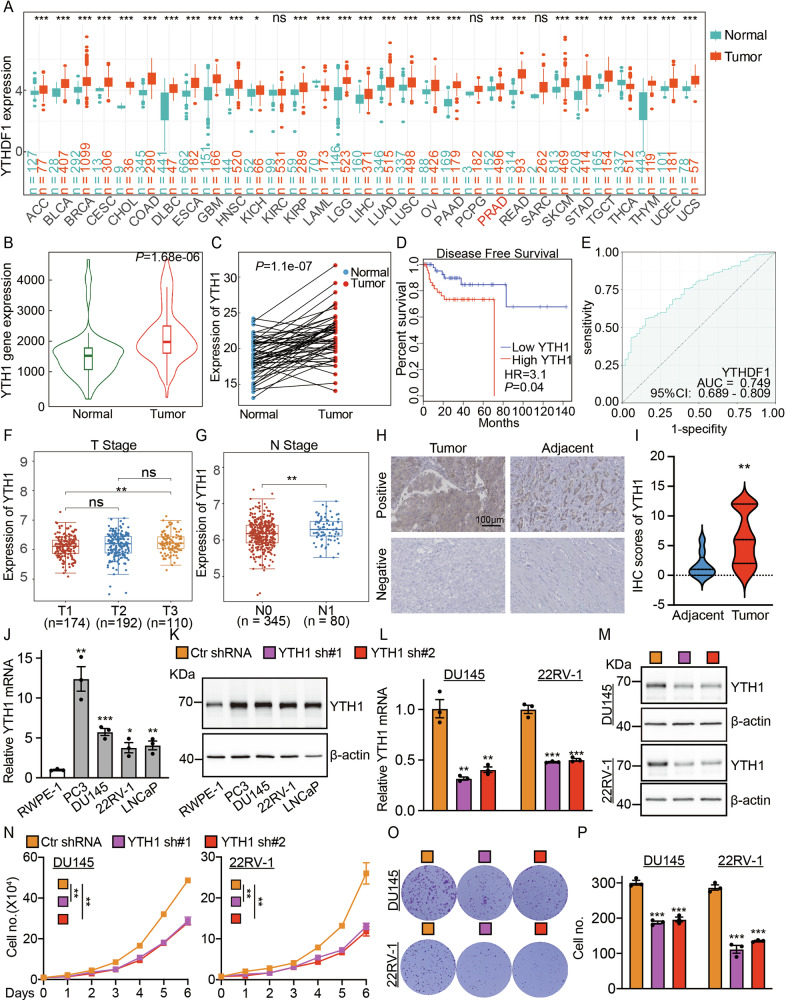
YTHDF1 is highly expressed in prostate cancer. **A** The expression levels of YTHDF1 across various tumor types were analyzed utilizing data from the TCGA database. **B** Analysis from the TNMplot website indicated that YTHDF1 expression in prostate cancer tissues was elevated. **C**. A matched expression analysis of prostate cancer patients using the TCGA database revealed that YTHDF1 was significantly overexpressed in prostate cancer. **D** Prostate cancer patients exhibiting high YTHDF1 expression were associated with a poorer prognosis. **E** The Receiver Operating Characteristic (ROC) curve analysis of YTHDF1 revealed an Area Under the Curve (AUC) value of 0.749. **F**, **G** YTHDF1 expression positively correlates with elevated tumor stages. **H**, **I** Tissue microarray staining demonstrated a markedly high expression of YTHDF1 in cancerous tissues. **J**, **K** YTHDF1 exhibited high expression in prostate cancer cell lines at both the mRNA (**J**) and protein (**K**) levels. **L**, **M** Assessment of YTHDF1 knockdown efficiency in DU145 and 22RV-1 cell lines was conducted, with (**L**) representing mRNA levels and (**M**) representing protein levels. **N** The knockdown of YTHDF1 resulted in the inhibition of proliferation in prostate cancer cell lines. **O-P** The knockdown of YTHDF1 led to a reduction in colony formation efficiency, with (**P**) providing the statistical analysis of the results observed in (**O**). Means ± SEM, *P < 0.05; **P < 0.01; ***P < 0.001; *t*-test. Ctr =Control shRNA or scrambled shRNA; YTH1 = YTHDF1; sh=shRNA; sh#1=shRNA#1; sh#2=shRNA#2.

By a comprehensive analysis using the TCGA (The Cancer Genome Atlas) database, we observed that YTHDF1 is highly expressed across various tumor types, including prostate cancer (Figs. 1A, S1H). Subsequent analysis revealed that YTHDF1 expression is significantly elevated in prostate cancerous tissues compared to control (adjacent or paracancerous) tissues (Fig. 1B, C). Conversely, the expression levels of the other two YTHDF family members, YTHDF2 and YTHDF3, were reduced in prostate cancer (Fig. S1A, B). Furthermore, the elevated expression of YTHDF1 demonstrated a significant positive correlation with patient's poorer prognosis (Fig. 1D), whereas YTHDF2 and YTHDF3 did not exhibit a significant correlation with patient's prognosis (Fig. S1C, D). The receiver operating characteristic (ROC) curve analysis demonstrated an AUC value of 0.749 for YTHDF1, suggesting its potential as an independent biomarker for prostate cancer (Figs. 1E, S1E, F). Furthermore, YTHDF1 correlates with the stages of prostate cancer (Fig. 1F, G), while it did not exhibit a significant association with prostate cancer metastasis (Fig. S1G). Concurrently, our tissue microarray analysis revealed that YTHDF1 was significantly overexpressed in prostate cancer tissues (Fig. 1H, I). Our findings demonstrated significant overexpression of YTHDF1 in prostate cancer cell lines PC3, DU145, 22RV-1, and LNCaP, relative to the normal prostate cells RWPE-1 (Fig. 1J, K). The findings suggest that YTHDF1 may play a role in prostate cancer pathogenesis. We created YTHDF1 knockdown cell lines in DU145 and 22RV-1, demonstrating that YTHDF1 knockdown markedly reduced tumor cell proliferation (Fig. 1L–P).

### YTHDF1 regulates tumor cell cycle transition

**Fig. 2:**
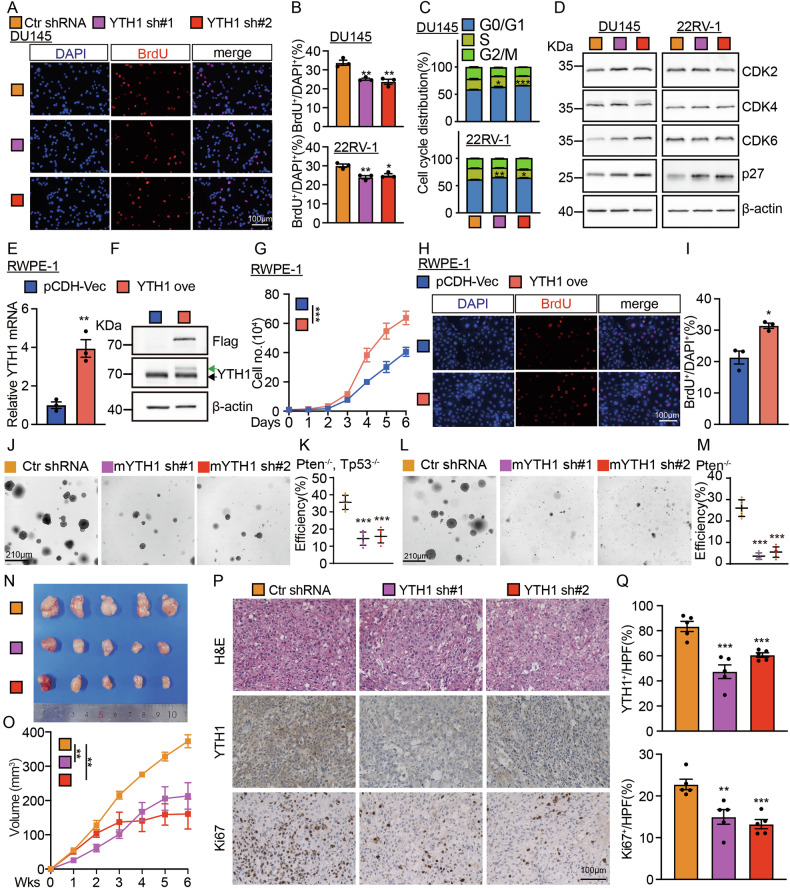
YTHDF1 promotes the cell cycle transition in PCa. The knockdown of YTHDF1 in DU145 cells significantly decreased the incorporation efficiency of BrdU, with (**B**) presenting the statistical analysis of (**A**). Scale bar = 100 μm. **C** Statistic results for the FACS analysis of YTHDF1 knockdown leading to an increased number of cells in the G0/G1 phase. **D** Immunoblot analysis demonstrated the changes in the expression of cell cycle-related proteins, including CDK2, CDK4, CDK6, and p27, following YTHDF1 knockdown. The overexpression of YTHDF1 in RWPE-1 cells was assessed, with (**E**) analyzing mRNA levels and (**F**) analyzing protein levels. The green arrow indicates exogenous YTHDF1-flag, while the black arrow denotes endogenous YTHDF1. **G** The overexpression of YTHDF1 in RWPE-1 cells enhances tumor cell growth. The overexpression of YTHDF1 in RWPE-1 cells increases BrdU incorporation efficiency, with (**I**) representing the statistical analysis of (**H**). Scale bar = 100 μm. **J**, **K** Organoid images derived from prostatic *Pten* and *Tp53* knockout mice (*Pten*^−/−^; *TP53*^−/−^), with or without mouse mYTHDF1 knockdown. The statistical result was presented in (**K**) for (**J**). Organoid images derived from prostatic Pten knockout mice (Pten^-/-^), with or without YTHDF1 knockdown. The statistical result was presented in (**M**) for (**L**). **N** Images of xenograft tumors. **O** Silencing YTHDF1 resulted in reduced tumor volume. Immunohistochemical (IHC) analysis of xenograft tumors, including H&E staining and expressions of YTHDF1 and Ki67, with (**Q**) representing the statistical analysis of (**P**). YTH ove = YTHDF1 overexpression. Means ± SEM, *P < 0.05; **P < 0.01; ***P < 0.001; *t*-test.

Given that YTHDF1 facilitates the proliferation of prostate cancer cells, we uncovered that silencing YTHDF1 effectively disrupted the cell cycle transition by BrdU incorporation assay (Figs. 2A, B, S2A). Flow cytometry analysis indicated that YTHDF1 knockdown caused cell cycle arrest in the G0/G1 phase (Figs. 2C, S2B). The detection of the key regulators involved in G0/G1 phase cell cycle transition revealed a significant upregulation of p27 protein [21], whereas CDK2, CDK4, and CDK6 proteins exhibited no significant alterations (Fig. 2D). Moreover, we overexpressed YTHDF1 in human normal prostate RWPE-1 cells and assessed the potential oncogenic effect, and showed that YTHDF1 forced expression enhanced cell proliferation (Fig. 2E–I). After confirming that YTHDF1 promotes tumor cell proliferation in vitro, we evaluated its effect in vivo. Initially, we utilized mouse prostate cancer organoids [22], and the findings demonstrated that knockdown of mouse YTHDF1 (mYTHDF1) markedly reduced the clonogenic formation of *Pten*- or/and *TP53*-deficient organoids (Figs. 2J–M, S2C–D). Concurrently, experiments applying xenograft tumor formation in nude mice showed that the knockdown of YTHDF1 markedly retarded tumor growth in vivo (Fig. 2N). The xenograft tumor phenotype was further evidenced by a reduction in tumor volume after YTHDF1 knockdown (Fig. 2O), alongside a significant decrease in the Ki67 staining positive rate, as determined by immunohistochemistry (IHC) staining (Fig. 2P–Q). After evaluating YTHDF1’s role in advancing tumor cell cycle progression, we examined its known targets, CDK2 and CDK4, in non-small cell lung cancer using RNA Binding Protein Immunoprecipitation Assay (RIP) [23]. The results indicated that YTHDF1 does not bind to the mRNAs of CDK2 and CDK4 in prostate cancer cells. Given the observed alteration in the proteins of cyclin p27, we did not observe that YTHDF1 bound to p27 mRNA (Fig. S2E), indicating that YTHDF1 promotes tumor cell proliferation via a novel target in prostate cancer.

### YTHDF1 increases the translational efficiency of RNF7 transcript

**Fig. 3:**
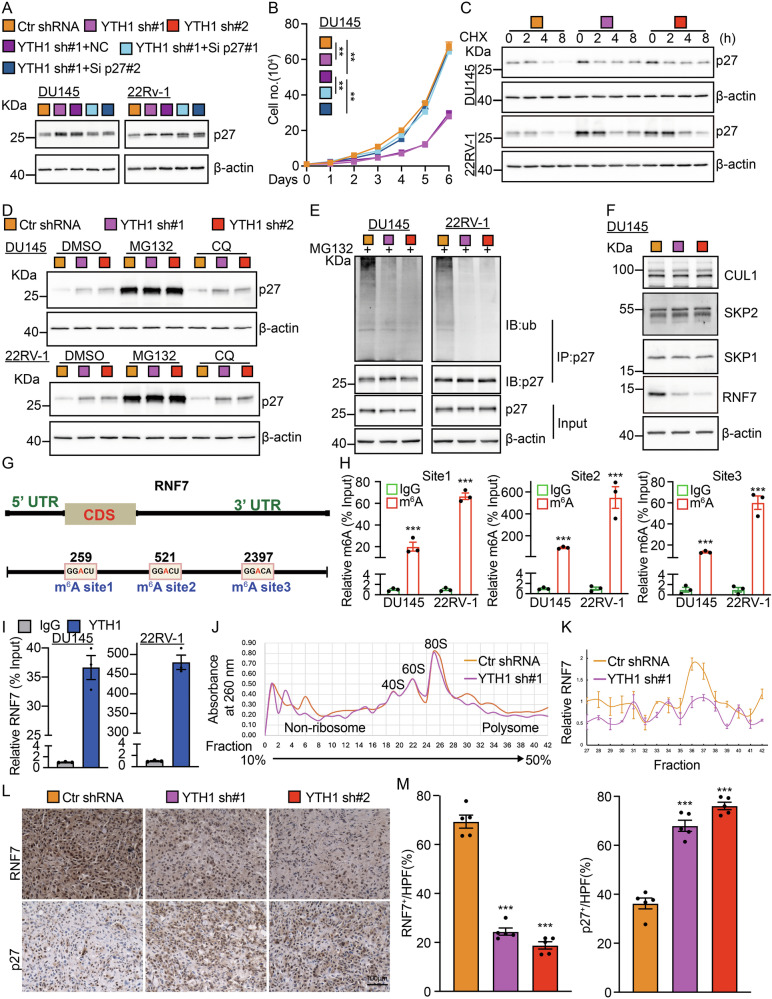
YTHDF1 decreases p27 protein stability. **A** The p27 proteins were detected in DU145 and 22RV-1 following YTHDF1 depletion with or without p27 knockdown using immunoblot. **B** p27 knockdown overcame the inhibitory effect upon YTHDF1 knockdown by cell growth assay. **C** Cell lysates from indicated cell lines, treated with cycloheximide (CHX: 100 µg/mL), were analyzed via immunoblot using indicated antibodies. **D** Total p27 protein levels were upregulated by the proteasome inhibitor MG132, but not by the lysosomal inhibitor chloroquine (CQ), examined by immunoblot. **E** The ubiquitination of p27 following YTHDF1 knockdown was analyzed in the specified cell lines using immunoblot with indicated antibodies. **F** The protein expression levels of several E3 ligases, including CUL1, SKP2, SKP1, and RNF7, were assessed in DU145 cells following YTHDF1 knockdown. **G** Prediction of m6A modification sites on RNF7. **H** The m6A modification sites on RNF7 transcript in DU145 and 22RV-1 cells were identified using m6A-RIP using indicated primers. **I** YTHDF1-RIP analysis demonstrates that YTHDF1 is capable of binding to RNF7 mRNA. **J**, **K** Polysome profiling showed that the knockdown of YTHDF1 reduced RNF7 translation efficiency. **L**, **M** The IHC staining in xenograft tumors of nude mice showed that the positive staining signal of RNF7 was weakened, while the positive staining signal of p27 was enhanced after knocking down YTHDF1, (**M**) is the statistical result of (**L**). Scale bar = 100 μm. Si = siRNA. Means ± SEM, *P < 0.05; **P < 0.01; ***P < 0.001; *t*-test.

To further elucidate whether the upregulation of p27 is the key factor inhibiting tumor cell proliferation following YTHDF1 knockdown, we conducted a subsequent knockdown of p27 in indicated cells already subjected to YTHDF1 knockdown and found that p27 knockdown countered the effect of YTHDF1 (Figs. 3A–B, S3A). Notably, there was no significant alteration in the RNA levels of p27 upon YTHDF1 knockdown (Fig. S3B). Considering that YTHDF1 does not bind to the p27 transcript, we hypothesized that YTHDF1 reduces p27 protein expression via other mediators at the post-transcriptional level. Consequently, cycloheximide (CHX) was introduced to the cells, revealing a marked enhancement in the stability of the

p27 protein within the YTHDF1 knockdown group (Figs. 3C, S3C). Furthermore, we uncovered that only the protein proteasome degradation signaling inhibitor MG132, but not the lysosomal inhibitor CQ (Chloroquine), was capable of reversing the decreased p27 proteins in both YTHDF1 overexpressing and knockdown cells, suggesting that YTHDF1 facilitates cell cycle progression by enhancing the ubiquitin degradation of the p27 proteins (Figs. 3D, S3H).

Therefore, we assessed the ubiquitination status of p27 in prostate cancer cells and observed a significant reduction in its ubiquitination level following YTHDF1 knockdown (Fig. 3E). We analyzed expressions of known p27-targeting E3 ubiquitin ligases, such as CUL1 (cullin 1), SKP1 (S-phase kinase associated-protein 1), and SKP2, in prostate cancer cells [24]. In YTHDF1 knockdown tumor cells, only RNF7 (RING Finger protein 7), also known as SAG (Sensitive to Apoptosis Gene), a crucial subunit of the RBX/ROC RING of E3 ubiquitin ligase essential for cancer cell growth [25–27], was significantly reduced compared to control cells. Meanwhile, CUL1, SKP1, and SKP2 levels remained unchanged (Figs. 3F, S3D), whereas RNF7 mRNAs remained largely unaltered (Fig. S3E–F). Importantly, we predicted three candidate m^6^A modification sites on the RNF7 mRNA using the m^6^A Atlas dataset, which was subsequently validated through m6A-RIP and YTHDF1-RIP experiments (Figs. 3G–I, S3G). The polysome profiling analysis further corroborated that silencing YTHDF1 reduces the translational efficiency of RNF7 (Fig. 3J–K). IHC staining of xenograft tumor tissues revealed decreased RNF7 and increased p27 levels in YTHDF1 knockdown groups compared to controls (Fig. 3L–M). Together, these findings substantiate the role of YTHDF1 in enhancing the translational efficiency of RNF7, thereby facilitating the ubiquitin-mediated degradation of the p27 proteins, and leading to robust tumor growth.

### RNF7 functions as an oncogene in prostate cancer

**Fig. 4:**
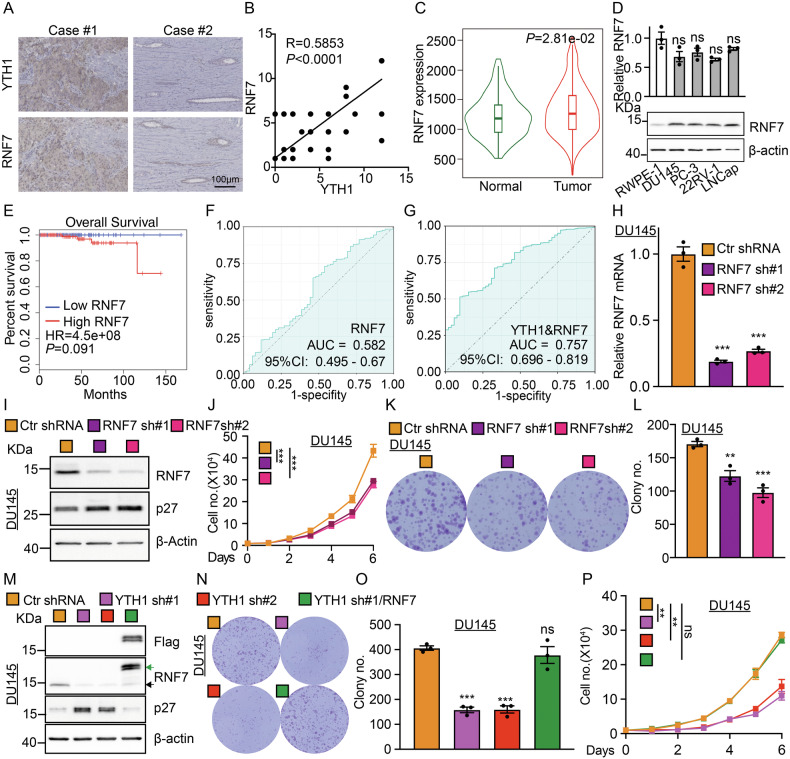
YTHDF1/RNF7/p27 axis promotes tumor growth. **A**, **B** The expression levels of YTHDF1 and RNF7 were analyzed using the serial tumor tissue sections from different PCa patients. (**B**) Is the statistic result for (**A**). Scale bar = 100 μm. **C** According to TNMplot analysis, no significant difference was observed in the RNA expression of RNF7 between prostate cancer tissues and normal tissues. **D** qRT-PCR (top) and Immunoblot (bottom) analysis were conducted to assess RNF7 RNA and protein expressions in prostate cancer cell lines. **E** The RNA expression of RNF7 is not strongly associated with the prognosis of patients with prostate cancer. **F** Receiver Operating Characteristic (ROC) curve analysis of RNF7 RNA yielded an Area Under the Curve (AUC) value of 0.582. **G** The AUC value of RNF7 and YTHDF1 was 0.757. RNF7 was knocked down in DU145 cells, indicated transcripts and proteins were examined by qRT-PCR (**H**) and immunoblot (**I**), respectively. **J** RNF7 knockdown in DU145 cells led to reduced tumor cell proliferation. **K**, **L** The knockdown of RNF7 in DU145 cells resulted in a decreased colony formation efficiency. (**L**) is the statistical analysis result for (**K**). **M** Validation of RNF7 overexpression in YTHDF1 knockdown DU145 cells by immunoblot with indicated antibodies. **N**, **O** RNF7 overexpression in DU145 cells counteracted the suppression of colony formation induced by YTHDF1 knockdown in prostate cancer cell lines, with (**O**) representing the statistical analysis of (**N**). **P** RNF7 overexpression in DU145 cells mitigates the reduction in cell proliferation following YTHDF1 knockdown in prostate cancer cell lines. YTH1 sh#1/RNF7 = YTHDF1 shRNA#1 + RNF7 overexpression. Means ± SEM, *P < 0.05; **P < 0.01; ***P < 0.001; *t*-test.

To validate the critical role of YTHDF1/RNF7/p27 in prostate cancer, we initially assessed RNF7 expression in a prostate cancer tissue microarray and observed a positive correlation between RNF7 and YTHDF1 proteins, without detecting significant change in RNF7 mRNAs (Fig. 4A–C). RNF7 protein levels, but not transcript levels, were found to be elevated in prostate cancer cell lines, compared to that in RWPE-1 (Fig. 4D). In alignment with the findings for YTHDF1, patients exhibiting high RNF7 expression were associated with a poorer prognosis (Fig. 4E). However, the AUC value for the combined analysis of YTHDF1 and RNF7 was 0.757, which represents a significant increase compared to the AUC of 0.582 for RNF7 alone. Meanwhile, there was no dramatic improvement over the AUC of 0.749 for YTHDF1, which further suggests that YTHDF1 may be involved in the translation process of the RNF7 protein (Figs.1E, 4F–G). We further performed RNF7 knockdown in prostate cancers and observed a significant inhibition of tumor cell proliferation (Figs. 4H–L, S4A–D). As expected, we overexpressed RNF7 in prostate cancer cell lines in combination with YTHDF1 knockdown, which counteracted the inhibitory effect of YTHDF1 knockdown on tumor cell proliferation (Figs. 4M–P, S4E–H).

### YTHDF1 knockdown promotes tumor cell sensitivity to chemotherapy

**Fig. 5:**
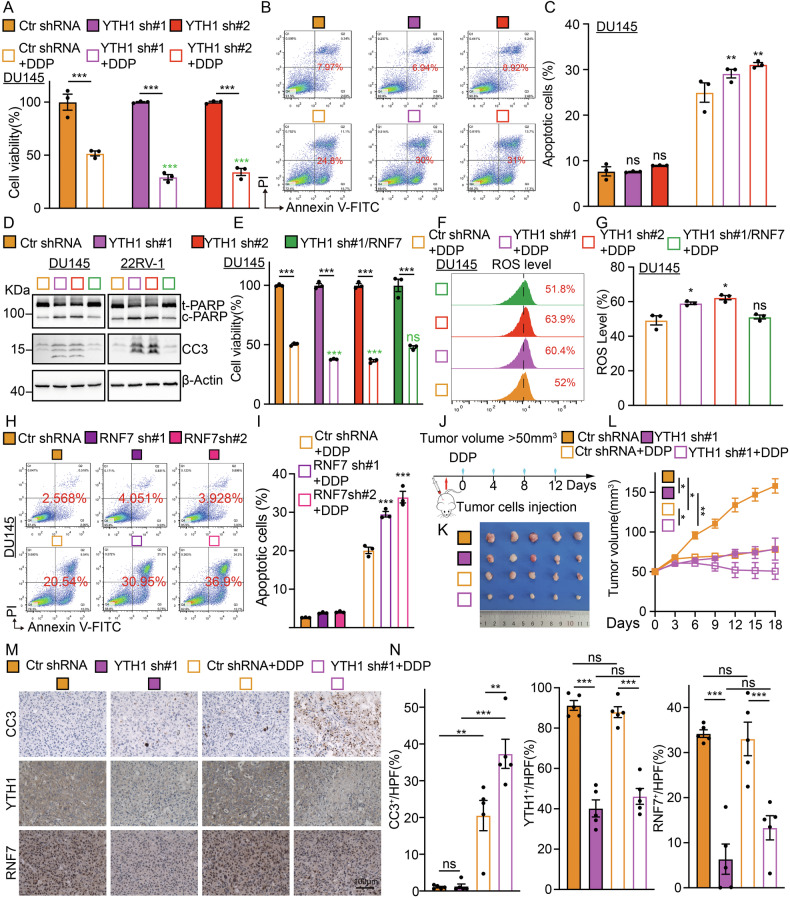
YTHDF1 knockdown promotes tumor cell sensitivity to DDP. **A** The addition of DDP to DU145 cells with YTHDF1 knockdown resulted in a significant reduction in cell survival, as assessed by the SRB assay. Green *P* values were quantified in DDP treatment groups. **B**, **C** In DU145 cells with YTHDF1 knockdown, DDP treatment led to increased cellular apoptosis, as determined by flow cytometry assay, with (**C**) presenting the statistical analysis of (**B**). **D** Immunoblot analysis was employed to examine the cellular apoptosis-related proteins, including PARP (t-PARP=total PARP and cleaved PARP=c-PARP) and CC3 (cleaved caspase 3) in YTHDF1 knockdown cells or with the overexpression of RNF7 treated with DDP (30 µM), indicated antibodies were used. **E** The overexpression of RNF7 in DU145 cells counteracted the suppression of cell viability following YTHDF1 knockdown and DDP administration. Green *P* values were quantified in DDP treatment groups. **F**, **G** RNF7 overexpression reduced reactive oxygen species (ROS) production, with (**G**) representing the statistical analysis of (**F**). **H**, **I** In DU145 cells with RNF7 knockdown, DDP treatment led to an increased cellular apoptosis, as determined by flow cytometry, with (**I**) presenting the statistical analysis of (**H**). **J**, **L** Schematic diagram shows the experimental strategy for DDP (7 mg/kg) treatment in nude mice (**J**). In detail, after xenograft tumors reached about 50 mm^3^ in size, mice were injected with DDP intraperitoneally every 4 days. At the end of the experiment, representative xenograft tumor images (**K**) and tumor volumes (**L**) were recorded for the indicated groups. **M**, **N** Representative IHC staining of YTHDF1, RNF7, and CC3 for indicated xenograft tumors with or without DDP treatment (**M**). The quantification data are included (**N**). Scale bar = 100 μm. Means ± SEM, *P < 0.05; **P < 0.01; ***P < 0.001; *t*-test.

To explore the clinical application of YTHDF1 in prostate cancer, we decided to examine whether inhibiting YTHDF1 would be able to increase tumor cell sensitivity to chemotherapy drug. The molecular mechanism of the antitumor effect of first-line chemotherapy drug cisplatin (DDP) mainly involves DNA damage-induced ROS (reactive oxygen species) dependent cellular apoptosis [28]. As expected, DDP treatment upon YTHDF1 knockdown significantly reduced tumor cell viability (Figs. 5A, Fig. S5A). Flow cytometry analysis verified that knocking down YTHDF1 markedly increased cellular apoptosis (Figs. 5B–C, S5B–C), evidenced by increased both cleaved PARP and cleaved caspase3 after DDP treatment and the overexpression of RNF7 can restore the levels of cleaved PARP and cleaved caspase3 (Fig. 5D). Prior research has shown RNF7’s involvement in ROS clearance [29]. Our findings confirmed that YTHDF1 depletion significantly elevated intracellular ROS levels induced by DDP treatment, which was subsequently reduced by RNF7 overexpression (Figs. 5E–G, S5D–H). Consistently, we observed similar synergistic effects in RNF7 knockdown cells upon DDP treatment (Figs. 5H–I, S5I–J). The above findings were validated in vivo using a xenograft tumor model, and we showed that YTHDF1 knockdown retarded tumor growth upon DDP treatment (Figs. 5J–L, S5K). IHC staining revealed a significant increase in CC3 and a marked decrease in Ki67 in YTHDF1 knockdown groups compared to the control group (Figs. 5M–N, S5L–M).

### RNF7 serves as a potential therapeutic target in prostate cancer

**Fig. 6:**
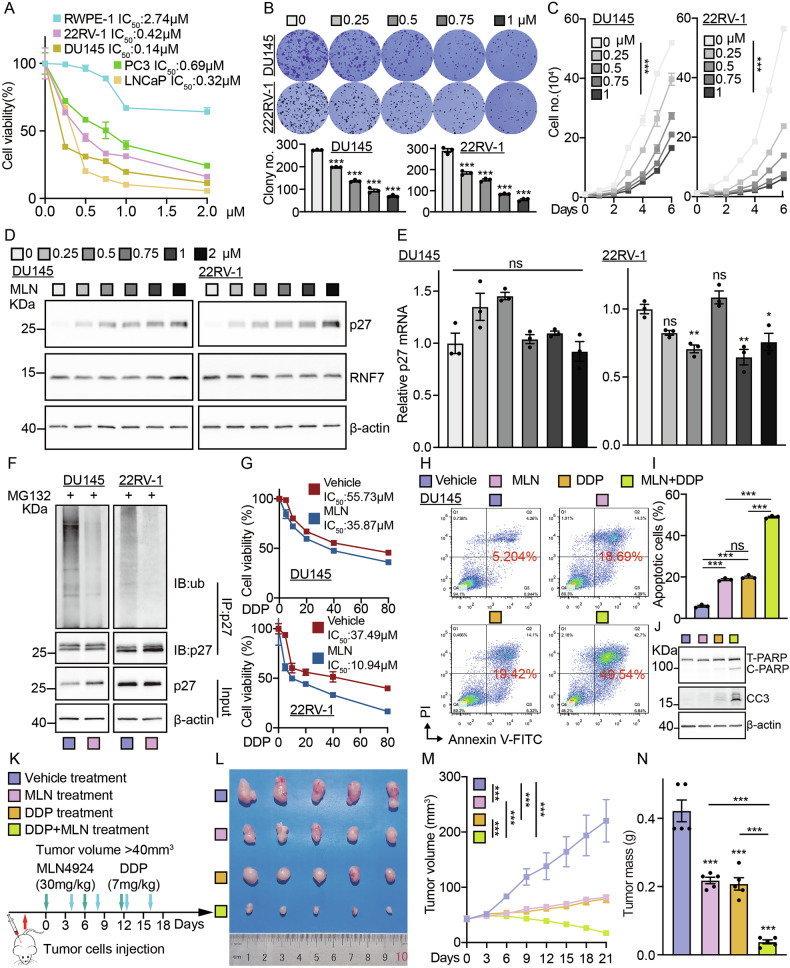
Neddylation inhibitor MLN4924 repressed tumor growth. **A** The IC_50_ of indicated cell lines treated with different concentrations of MLN4924. (MLN = MLN4924). **B** Representative images for the colony assay in DU145 and 22RV-1 cells treated with indicated dosages of MLN. **C** The growth curve of DU145 and 22RV-1 cells treated with indicated dosages of MLN. The proteins (**D**) and mRNAs (**E**) of p27 and RNF7 in DU145 cells treated with indicated dosages of MLN were examined by immunoblot and qRT-PCR, respectively. **F** The ubiquitination of p27 after being treated with MLN (0.2 µM) was analyzed in the specified cell lines using immunoblot with indicated antibodies. **G** The cell viability assay for indicated cells treated with vehicle or MLN (0.2 μM) in combination with indicated dosages of DDP. **H**–**J** DU145 cells treated with or without MLN (0.2 µM) in combination with or without DDP (30 µM) were examined by flow cytometry assay (**H**, **I**) and immunoblot (**J**). **I** is the statistical result for (**H**). **K** The schematic picture of the xenograft mouse model treated with vehicle, MLN (60 mg/kg), DDP (7 mg/kg), or MLN and DDP. **L**–**N** Representative xenograft tumor mass images (**L**), tumor volume (**M**), and tumor masses (**N**). Means ± SEM, *P < 0.05; **P < 0.01; ***P < 0.001; *t*-test.

To examine whether inhibiting RNF7 exhibits similar clinical application potential as YTHDF1, we used MLN4924, a small molecule inhibitor of NEDD8-Activating Enzyme (NAE), which inactivates RNF7 by blocking cullin neddylation [30]. We revealed that MLN4924 significantly decreased tumor cell viability (Fig. 6A–C). In line with previous results, the addition of MLN4924 to tumor cells significantly inhibited tumor cell proliferation and enhanced the stability of p27 proteins but not transcripts in both concentration and time gradients in a ubiquitination-dependent manner (Figs. 6D–F, S6A–B). Furthermore, MLN4924 in combination with DDP treatment robustly increased cellular apoptosis (Figs. 6G–J, S6C–E). In addition, the xenograft tumor formation experiment verified that MLN4924 administration repressed tumor growth and enhanced tumor cell sensitivity to DDP (Fig. 6K–N), as evidenced by the increased positive p27 and CC3, while decreasing Ki67, IHC staining ratios with or without DDP treatment (Fig. S6F–G). In summary, the above data suggest that neddylation inhibitor MLN4924 could be further developed to treat prostate cancer patients in the future.

## Discussion

YTHDF1, one member of the YTH domain-containing proteins recognizing N6-methyladenosine (m6A) modified mRNAs, plays pivotal roles regulating multiple mRNA translation efficiency and stability during various oncogenic processes [14]. Our previous findings have demonstrated that YTHDF1 facilitates tumor proliferation by stabilizing the translation of CDK2, CDK4, and CyclinD1 on the one hand, on the other hand, it enhances the translation of Keap1, leading to the degradation of Nrf2, thereby sensitizing tumor cell response to cisplatin treatment [11]. YTHDF1, induced by HIF-1α, promotes hypoxia-induced autophagy and hepatocellular carcinoma malignancy through m6A-modified ATG2A and ATG14 dependency [31]. Moreover, YTHDF1 has been shown to augment the translation of methylated HINT2 mRNA, thereby suppressing the malignant progression of ocular melanoma [32]. In addition, YTHDF1 escalates the translation of ARHGEF2, activating RhoA signaling, thereby driving tumorigenesis and metastasis in colorectal cancer [33]. The above findings highlight the multifaceted and context-dependent roles of YTHDF1 in various cancer types. Nevertheless, the critical YTHDF1 downstream m6A-modified factor regulating tumor progression is likely modulated by the tumor microenvironment and genomic landscape. Therefore, elucidating the functional dynamics and downstream targets of YTHDF1 in prostate cancer is imperative for advancing clinical precision oncology.

**Fig. 7:**
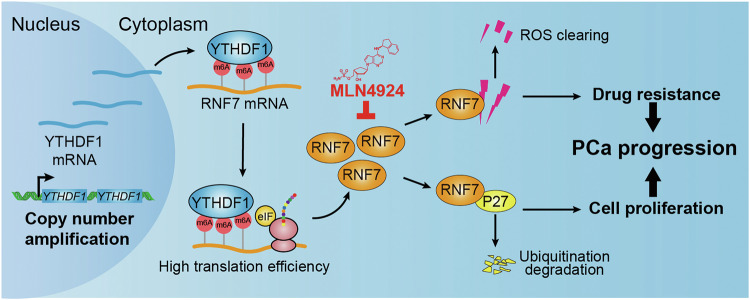
YTHDF1/RNF7/p27 axis promotes prostate cancer progression. YTHDF1 is highly expressed in prostate cancer, and its high expression correlates with poorer clinical outcome. YTHDF1 increases tumor cell proliferation, migration, and xenograft tumor formation abilities via inducing p27 protein stabilities depending on proteosome degradation signaling. Candidate p27-targeting E3 ubiquitin ligases screening identified RNF7 as the direct downstream target for YTHDF1 in an m6A-dependent manner. The subsequent high translation of RNF7 results in the efficient degradation of the cell cycle inhibitor p27 and malignant tumor cell growth. In addition, YTHDF1 or RNF7 increased the sensitivity to chemotherapy drug cisplatin (DDP) by inducing cellular apoptosis. Most importantly, neddylation inhibitor MLN4924 repressed PCa progression both in vitro and in vivo.

Consistent with former findings, we unveiled that abnormal amplification of the *YTHDF1* copy number leads to its overexpression in prostate cancer [11, 34, 35]. The ROC specificity of YTHDF1 in prostate cancer is close to 0.8, indicating its potential as an independent prognostic factor for patients with PCa. Our study reveals that YTHDF1 boosts the translation efficiency of E3 ligase RNF7, which in turn accelerates the degradation of the cell cycle inhibitor p27, promoting malignant tumor cell growth in vitro and in vivo. Interestingly, we uncovered that YTHDF1 forced overexpression in normal prostate cells RWPE-1 upregulates RNF7 expression, enhancing malignant cell proliferation and indicating the oncogenic role of the YTHDF1/RNF7 axis in PCa. In line with previous finding that RNF7 knockdown sensitizes tumor cell response to radiotherapy by increasing cellular apoptosis [36], we corroborated that YTHDF1 or RNF7 knockdown increased DDP-induced cellular apoptosis in prostate cancer cells (Fig. 7). Different from our previous study, we showed that YTHDF1/Keap1/Nrf2 axis promotes, while YTHDF1/RNF7/p27 inhibits, tumor cell sensitivity to DDP in NSCLC and PCa, respectively [11], suggesting that YTHDF1 regulates tumor cell fate in different cellular context, although YTHDF1 was mostly upregulated in pan-cancers.

Previous studies have shown that SAG/RNF7 is rich in cysteine and can inhibit apoptosis by reducing intracellular levels of reactive oxygen species (ROS) [37]. In renal cell carcinoma, RNF7 activates the JAK/STAT3 signaling pathway by inducing SOCS1 ubiquitination, leading to reduced cellular apoptosis [26]. Recent studies also showed that downregulation of RNF7 leads to inactivation of the PI3K/AKT signaling pathway, which suppresses cell proliferation while inducing apoptosis in glioma [27]. Our findings are consistent with previous studies, we showed that knockdown of YTHDF1 inhibits RNF7 expression, resulting in abnormal ROS accumulation upon DDP treatment in prostate cancer, while overexpression of RNF7 can reverse this effect.

We assessed the clinical potential of YTHDF1 in prostate cancer treatment by evaluating the therapeutic efficacy of the neddylation inhibitor MLN4924 using both in vitro and in vivo prostate cancer models. We found that MLN4924 treatment markedly sensitizes tumor cells to chemotherapeutic agent cisplatin. The efficacy of MLN4924 in repressing tumor progression has been validated in various cancers, including liver cancer [38], gastric cancer [39], and leukemia [40], indicating its promising clinical prospects in prostate cancer in the future (Fig. 7).

Recent research indicates that immune checkpoint blockade (ICB) therapy effectively treats advanced human cancers. However, only a small fraction of prostate cancer patients benefit from immunotherapy compared to other malignancies, suggesting an urgent need to develop novel strategies [41]. Prostate cancer, characterized as an “immune cold” tumor, exhibits minimal T-cell infiltration and limited response to checkpoint inhibitors [42]. Furthermore, YTHDF1-associated genes have been identified to be involved in immune response, antigen processing, and presentation [43]. YTHDF1 influences tumor antigen cross-presentation in dendritic cells and the cross-priming of CD8^+^ T cells [44], whereas its knockdown boosts the antitumor activity of specific CD8^+^ T cell populations [45]. These results suggest that targeting YTHDF1 may transform prostate cancer from an “immune cold” to an “immune hot” tumor, thereby increasing sensitivity to ICB therapies. In conclusion, our findings emphasize the critical role of YTHDF1 in the progression of prostate cancer and suggest its potential as a therapeutic target for future clinical interventions.

## Supplementary information

SUPPLEMENTAL MATERIAL

## Data Availability

In this study, the pan-cancer datasets and the prostate cancer datasets are available in the TCGA database (https://portal.gdc.cancer.gov). Some normal tissue sample datasets are available in the GTEx database (https://www.gtexportal.org/).
