# Supplementary material for: YTHDF1/RNF7/p27 axis promotes prostate cancer progression
Source: Cell Death Dis. 2025 Apr 18;16(1):314. doi: 10.1038/s41419-025-07648-3 (PMC12008233; doi:10.1038/s41419-025-07648-3)
Supplement: Supplementary file 1 — SUPPLEMENTAL MATERIAL [file 41419_2025_7648_MOESM1_ESM.docx]

Supplementary Materials for

**YTHDF1/RNF7/p27 axis promotes prostate cancer progression**

Yulin Shi^1,2,3,#^, Baiyang Liu^1,2,#^,Yong Zhang^4,#^, Sen Zhao^2,#^, Li Zuo^5^, Jun Pu^3^, Haoqing Zhai^2^, Dengcai Mu^2^, Jia Du^2^, Yan Cheng^1,*^, Cui-Ping Yang^6,7,^*, Yongbin Chen^1,2,^*

‡ These authors contributed equally to this work.

*Correspondence should be addressed to Y.B.C (email: [ybchen@mail.kiz.ac.cn](mailto:ybchen@mail.kiz.ac.cn)); C.P.Y (email: cuipingyang@sjtu.edu.cn); Y. C (email: cy20100323@163.com)

**This file includes:**

Supplementary Figures S1 to S6

Supplementary Tables S1 to S2

Uncropped Western blot

**Supplementary Figures and Figure legends**


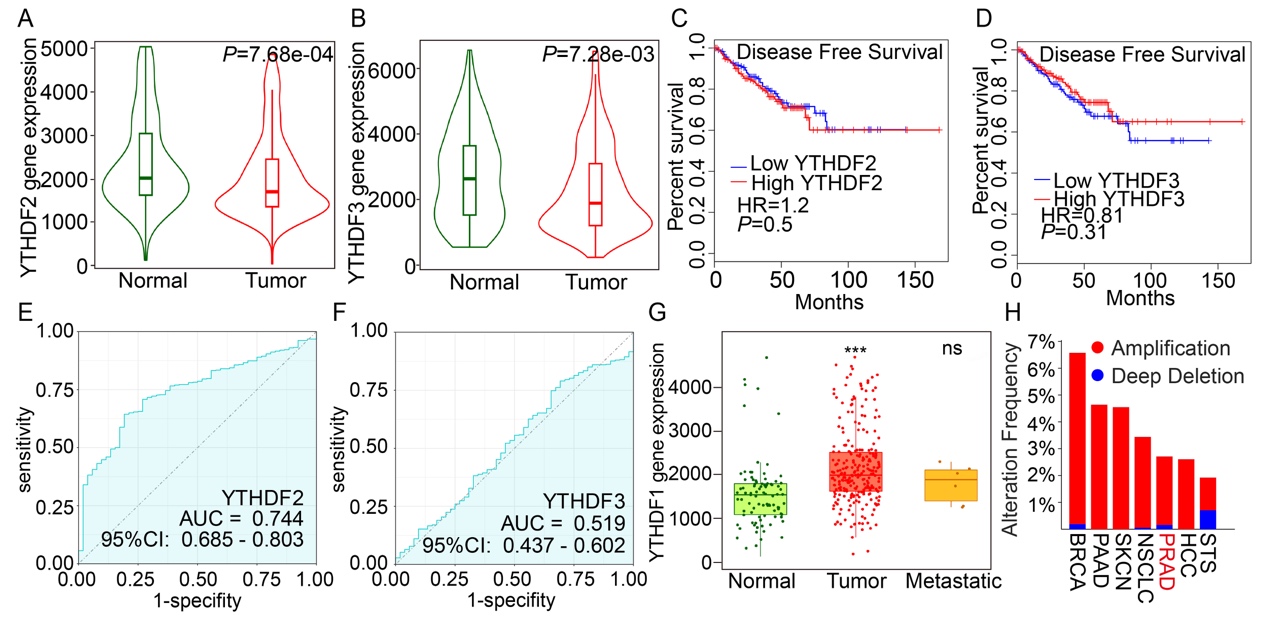


**Fig S1. YTHDF1 is highly expressed in prostate cancer.**

**A-B** TNMplot analysis revealed decreased expression in YTHDF2 (**A**) and YTHDF3 (**B**) expressions between prostate cancer tissues and normal tissues. **C-D** YTHDF2 (**C**) and YTHDF3 (**D**) showed no significant correlation with patient prognosis. **E-F** The receiver operating characteristic (ROC) curve analysis for YTHDF2 (**E**) and YTHDF3 (**F**) revealed an area under the curve (AUC) value of 0.744 and 0.519, respectively. **G** No significant association was observed between the expression level of YTHDF1 and tumor metastasis. **H** YTHDF1 is frequently found to be highly amplified in the genomic profiles of various tumors, Deletion: blue; amplification: red. Means ± SEM, **P* < 0.05; ***P* < 0.01; ****P* <0.001; *t*-test.


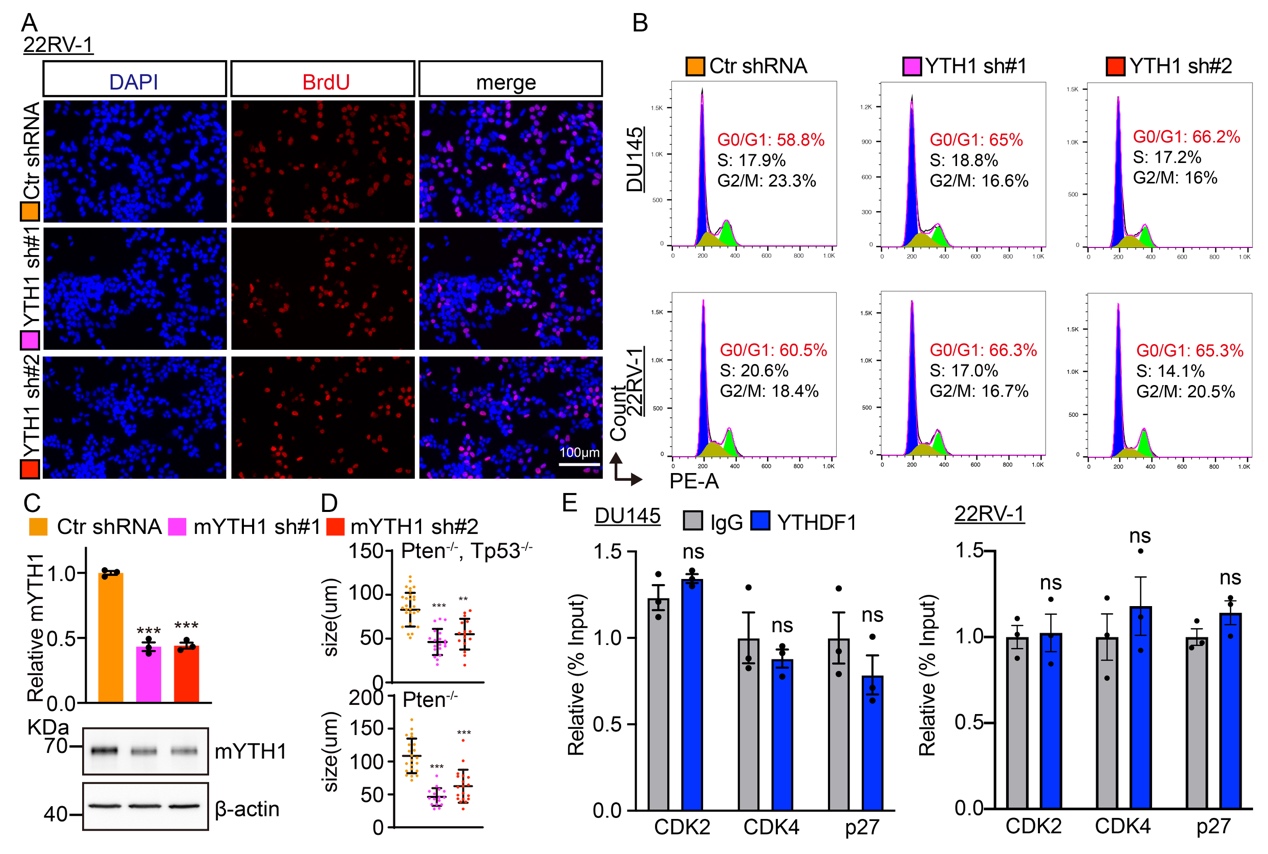


**Fig S2. YTHDF1 promotes the cell cycle transition in PCa.**

**A** The knockdown of YTHDF1 in 22RV-1 cells led to a significant reduction in BrdU incorporation efficiency. Scale bar=100μm. **B** Statistic result for the FACS analysis of YTHDF1 knockdown leading to an increased number of cells in the G0/G1 phase. **C** Evaluation of mouse YTHDF1 knockdown efficiency, with mRNA levels presented at the top and protein levels at the bottom. **D** The indicated organoid sizes were quantified. **E** RIP assays in DU145 and 22RV-1 cells demonstrated that YTHDF1 does not bind to CDK2, CDK4, or p27 mRNAs. Means ± SEM, **P* < 0.05; ***P* < 0.01; ****P* <0.001; *t*-test.


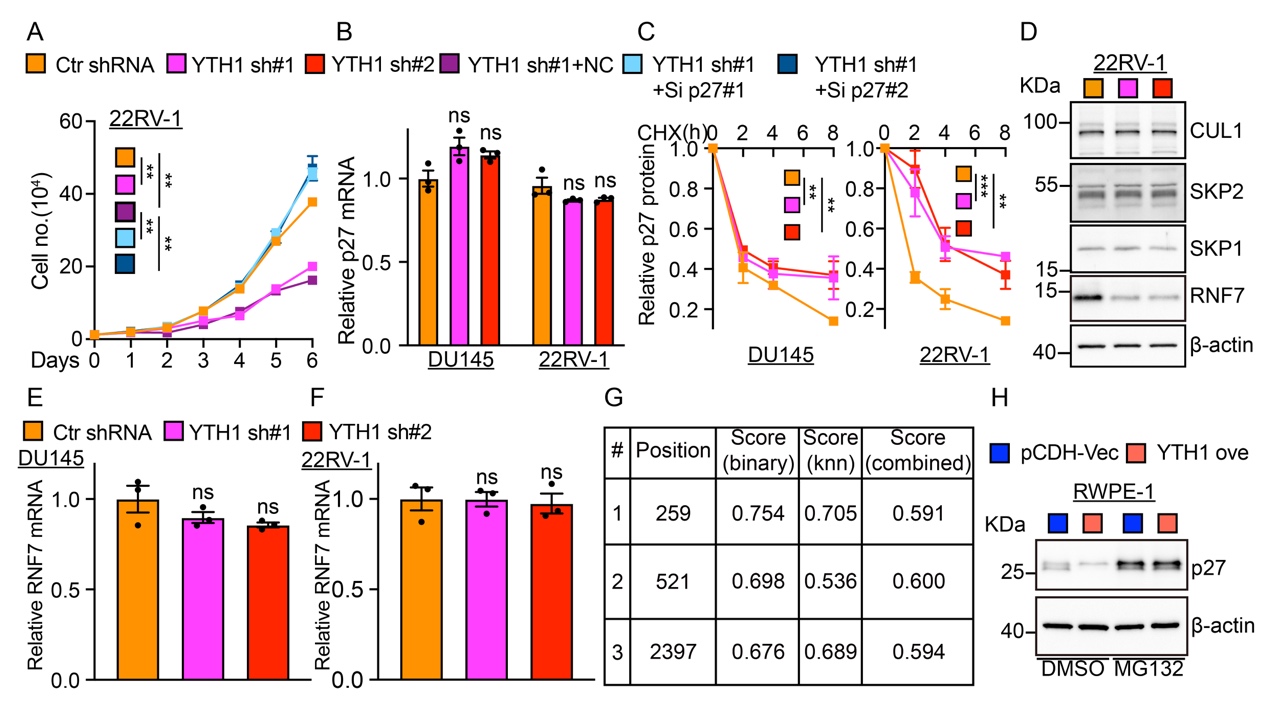


**Fig S3. YTHDF1 decreases p27 protein stability.**

**A** The knockdown of p27 exerts an inhibitory effect on the proliferation efficiency of 22RV-1 cells with YTHDF1 knockdown. **B** The mRNA expression level of p27 was quantified using qRT-PCR following YTHDF1 knockdown. **C** Quantitative analysis of protein levels as depicted in Figure 3C. **D** Protein expression levels of various E3 ligases, including CUL1, SKP2, SKP1, and RNF7, were assessed in 22RV-1 cells upon YTHDF1 knockdown. **E-F** The mRNA expression level of RNF7 was measured by qRT-PCR after YTHDF1 knockdown. **G** Prediction of m6A modification sites on RNF7 CDS. **H** Overexpression of YTHDF1 in RWPE-1 cells led to a decrease in total p27 protein levels, an effect that can be reversed by MG132 treatment. Means ± SEM, **P* < 0.05; ***P* < 0.01; ****P* <0.001; *t*-test.


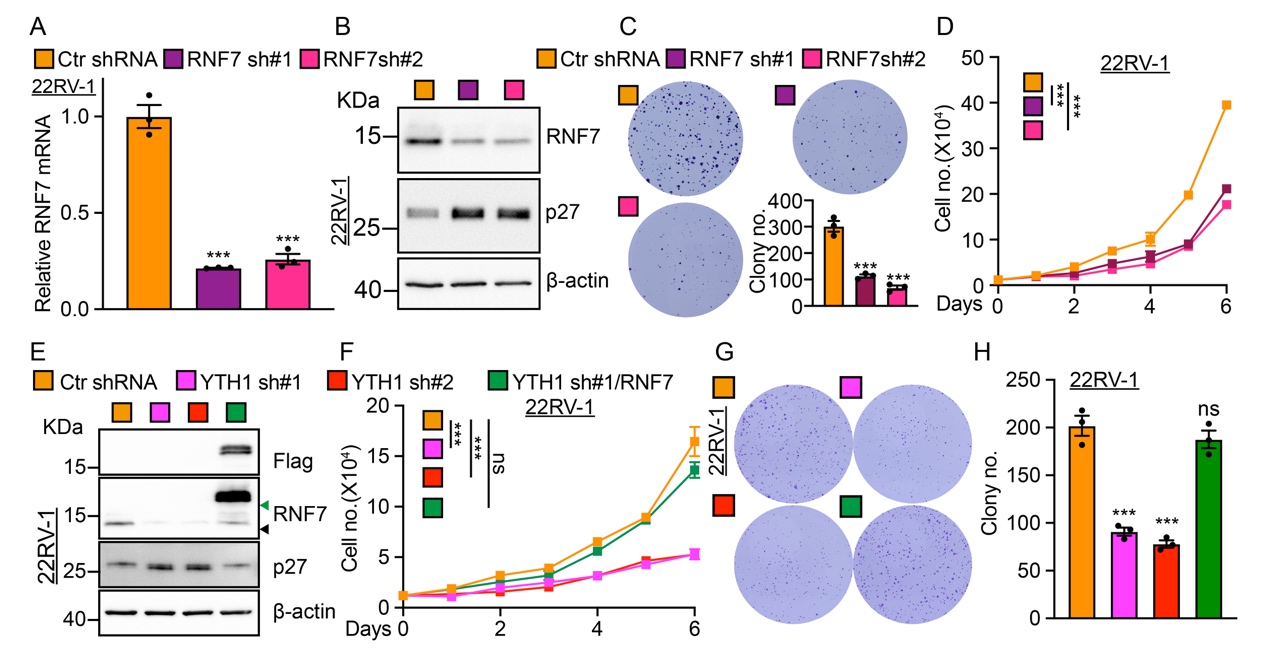


**Fig S4. YTHDF1/RNF7/p27 axis promotes tumor growth.**

**A-B** The knockdown efficiency of RNF7 and the detection of p27 in 22RV-1 cells were assessed at both the mRNA level (**A**) and the protein level (**B**). **C** The knockdown of RNF7 in 22RV-1 cells resulted in a reduction in the colony formation efficiency of prostate cancer cell lines, with statistical results presented. **D** The knockdown of RNF7 in 22RV-1 cells also inhibited the proliferation of prostate cancer cell lines. **E** The efficiency of YTHDF1 knockdown and RNF7 overexpression in 22RV-1 cells was evaluated and the expression of p27 was up-regulated after RNF7 overexpression. **F** The overexpression of RNF7 in 22RV-1 cells counteracted the suppression of cell proliferation following YTHDF1 knockdown in prostate cancer cell lines. **G-H** Overexpression of RNF7 in 22RV-1 cells reversed the inhibition of colony formation induced by YTHDF1 knockdown, with panel (**H**) presenting the statistical analysis of (**G**). Means ± SEM, **P* < 0.05; ***P* < 0.01; ****P* <0.001; *t*-test.


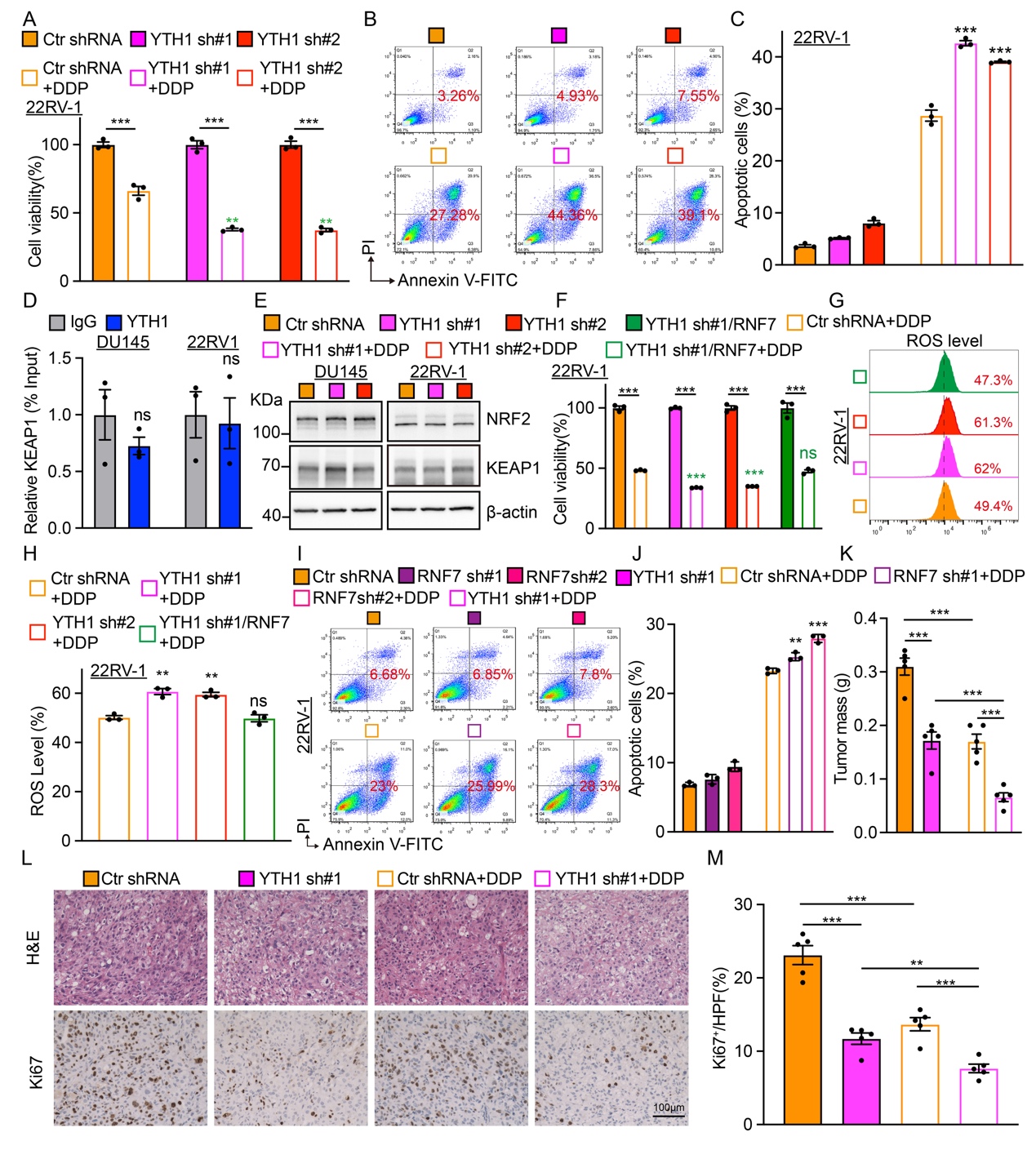


**Fig S5. YTHDF1 knockdown promotes tumor cell sensitivity to DDP.**

**A** The addition of DDP to 22RV-1 cells with YTHDF1 knockdown resulted in a significant reduction in cell survival rates, as assessed by SRB assay. Green *P* values were quantified in DDP treatment groups. **B-C** The introduction of DDP to 22RV-1 cells with YTHDF1 knockdown led to an increased apoptosis rate, as determined by flow cytometry, with (**C**) representing the statistical analysis of (**B**). **D** YTHDF1 RIP assay in DU145 and 22RV-1 cells demonstrated that YTHDF1 does not bind to KEAP1 mRNA. **E** The knockdown of YTHDF1 in DU145 and 22RV-1 cells was employed to investigate the alterations in NRF2 and KEAP1 protein levels. **F** The overexpression of RNF7 in 22RV-1 cells counteracted the reduction in cell survival rate induced by DDP (20µM) treatment following YTHDF1 knockdown in prostate cancer cell lines. **G-H** The overexpression of RNF7 in 22RV-1 cells mitigated the increase in reactive oxygen species (ROS) production observed in prostate cancer cell lines after YTHDF1 knockdown and subsequent DDP (20µM) treatment, with (**H**) representing the statistical analysis of **(G**). **I-J** The knockdown of RNF7 in 22RV-1 cells led to an elevated apoptosis rate following DDP (20µM) administration, with (**J**) providing the statistical analysis of (**I**). **K** Representative xenograft tumor masses were recorded for the indicated groups. **L-M** Representative H&E and IHC staining of Ki67 for indicated xenograft tumors with or without DDP (7mg/kg) treatment (**L**). The quantification data are included (**M**). Scale bar=100μm. Means ± SEM, **P* < 0.05; ***P* < 0.01; ****P* <0.001; *t*-test.


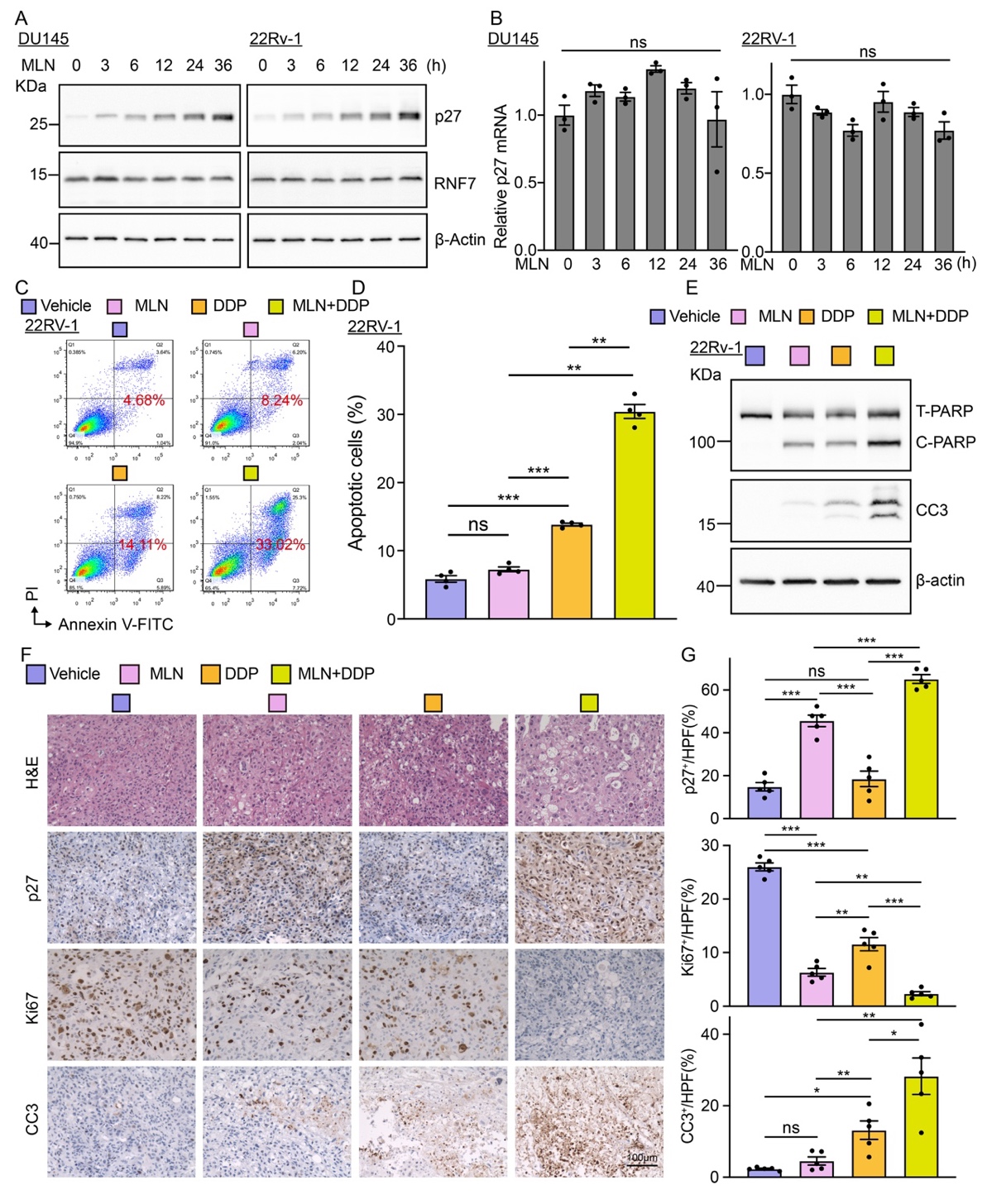


**Fig S6. Neddylation inhibitor MLN4924 repressed tumor growth.**

**A** The proteins of p27 and RNF7 in tumor cells treated with MLN were examined by immunoblot at the indicated time point. **B** The mRNAs of p27 in tumor cells treated with indicated dosages of MLN were examined by qRT-PCR. **C-E** 22RV-1 cells treated with or without MLN (0.1µM) in combination with or without DDP (20µM) were examined by flow cytometry assay (**C-D**) and immunoblot (**E**). (**D**) the statistical result for (**C**). **F-G** Representative IHC staining images of p27, Ki67, and CC3 in indicated xenograft tumor sections, (**G**) is the quantification data for (**F**). Scale bar=100μm. Means ± SEM, **P* < 0.05; ***P* < 0.01; ****P* <0.001; *t*-test.

**Supplementary Tables**

**Table S1. Antibodies used in this study.**

**Table S2. Primers and oligos used in this study.**
